# Cysteine-based molecular switches: a focus on KEAP1 and BACH1 in regulating the antioxidant response

**DOI:** 10.1007/s00204-026-04562-1

**Published:** 2026-10-08

**Authors:** Jialin Feng, Albena T. Dinkova-Kostova

**Affiliations:** 1 Division of Cancer Research, Jacqui Wood Cancer Centre, School of Medicine, University of Dundee, Ninewells Hospital and Medical School, James Arrott Drive, Dundee, DD1 9SY UK; 2 Department of Physiology, Pharmacology and Therapeutics, Johns Hopkins University School of Medicine, Baltimore, MD USA; 3 Department of Medicine, Johns Hopkins University School of Medicine, Baltimore, MD USA

**Keywords:** electrophile, NQO1, NRF2, omaveloxolone, RNS, ROS

## Abstract

Due to the chemical properties of their thiol groups and the surrounding amino acid environment, specific cysteines in proteins serve as the principal intracellular sensors for oxidants and electrophiles. In this review, we focus on the ubiquitin ligase substrate adaptor Kelch-like ECH-associated protein 1 (KEAP1) and the transcriptional repressor BTB and CNC homology 1 (BACH1) as representative cysteine-based molecular switches, both of which have critical functions in the cellular antioxidant response. KEAP1 is a protein endowed with a number of highly reactive cysteines, which can be covalently and preferentially modified by a wide array of reactive oxygen species, reactive nitrogen species and reactive electrophilic species, from endogenous as well as exogenous origin. Covalent cysteine modifications are also involved in the regulation of the localisation, stability and turnover of BACH1. Both KEAP1 and BACH1 antagonize the constitutively expressed transcription factor NF-E2 p45-related factor 2 (NRF2), the master regulator of the cellular antioxidant response, thus keeping the transcriptional activity of NRF2 low at homeostatic state. Cysteine modifications within KEAP1 and BACH1 render the repressor proteins inactive, resulting in rapid NRF2 activation that does not rely on de novo transcription of *NFE2L2*, the gene encoding NRF2. Consequently, NRF2 induces the transcriptional upregulation of a large network of genes encoding cytoprotective proteins, many of which have antioxidant functions, restoring the redox homeostasis.

## Introduction

The sulphur atom in the cysteine thiol (–SH) group is ionisable and can deprotonate to form a negatively charged thiolate (–S^−^), which is more nucleophilic and reactive. This deprotonation is favoured when the local environment stabilises the thiolate, for example, by the presence of nearby basic amino acids, hydrogen bond donors or positioning at the N-terminal end of a helix. These factors can lower the acid dissociation constant (pKa) of the thiol, facilitate thiolate formation and thereby enhance the reactivity of specific cysteines at physiological pH, which explains their disproportionate engagement in redox signalling (Lennicke and Cocheme 2021; Paulsen and Carroll 2013; Poole 2015). Cysteine residues exhibiting unusually high chemical reactivity, termed hyperreactive cysteines, are often functionally important sites involved in key biochemical processes, such as catalytic nucleophiles, redox switches or sites of regulatory post-translational modification. This striking correlation between cysteine hyperreactivity and functionality observed in native proteomes has inspired de novo protein design, where cysteine hyperreactivity serves as a predictive marker for protein function (Weerapana et al. 2010).

These principles align with evolutionary patterns. Firstly, cysteines show a bimodal conservation profile, where they are either highly conserved (functionally critical) or poorly conserved (context-dependent) (Marino and Gladyshev 2010). Secondly, cysteine residues tend to be localized in proximity to other cysteines, a feature known as ‘clustering’. This feature of cysteine distribution is more common in proteins expressed by organisms from harsh environments and is often characteristic of sites of metal-binding motifs or redox-sensitive loci that are subject to regulation by reversible disulfide bond formation (Beeby et al. 2005). Together, the chemistry and distribution patterns support the view that cysteine nucleophilicity has been evolutionarily selected and conserved to facilitate precise redox regulation through selective oxidative and electrophilic post-translational modifications (PTMs), such as *S*-glutathionylation, *S*-nitrosylation, and lipid aldehyde adduction, underscoring the sophisticated and selective nature of cysteine-mediated electrophilic sensing in cellular signalling (Weerapana et al. 2010). Functionally, this creates modular, reversible ‘switches’ that can be incorporated into diverse signalling networks and fine-tuned by cellular redox systems. Importantly, the cellular thioredoxin and glutaredoxin systems catalyse the reversal of many thiol PTMs, providing temporal control and the capacity to reset cysteine-based signalling switches (Lillig et al. 2008). It is therefore unsurprising that, although one of the least abundant amino acids, cysteine is frequently conserved within functional domains of proteins, and serves as a critical molecular switch that modulates protein function (Marino and Gladyshev 2010). In this review, we briefly summarize the mechanisms by which cysteines serve as sensors for reactive oxygen species (ROS), reactive nitrogen species (RNS), and reactive electrophilic species (RES). We then focus on the antioxidant response regulated by transcription factor NRF2, and the principal NRF2 negative regulator and cysteine-based sensor KEAP1. Finally, we discuss the importance of cysteine reactivity for the regulation of BACH1, a transcriptional repressor and NRF2 competitor, and give examples of RNS and RES that can dually inhibit KEAP1 as well as BACH1, converging on NRF2 activation.

### Cysteines as sensors for reactive oxygen and nitrogen species

**Fig. 1:**
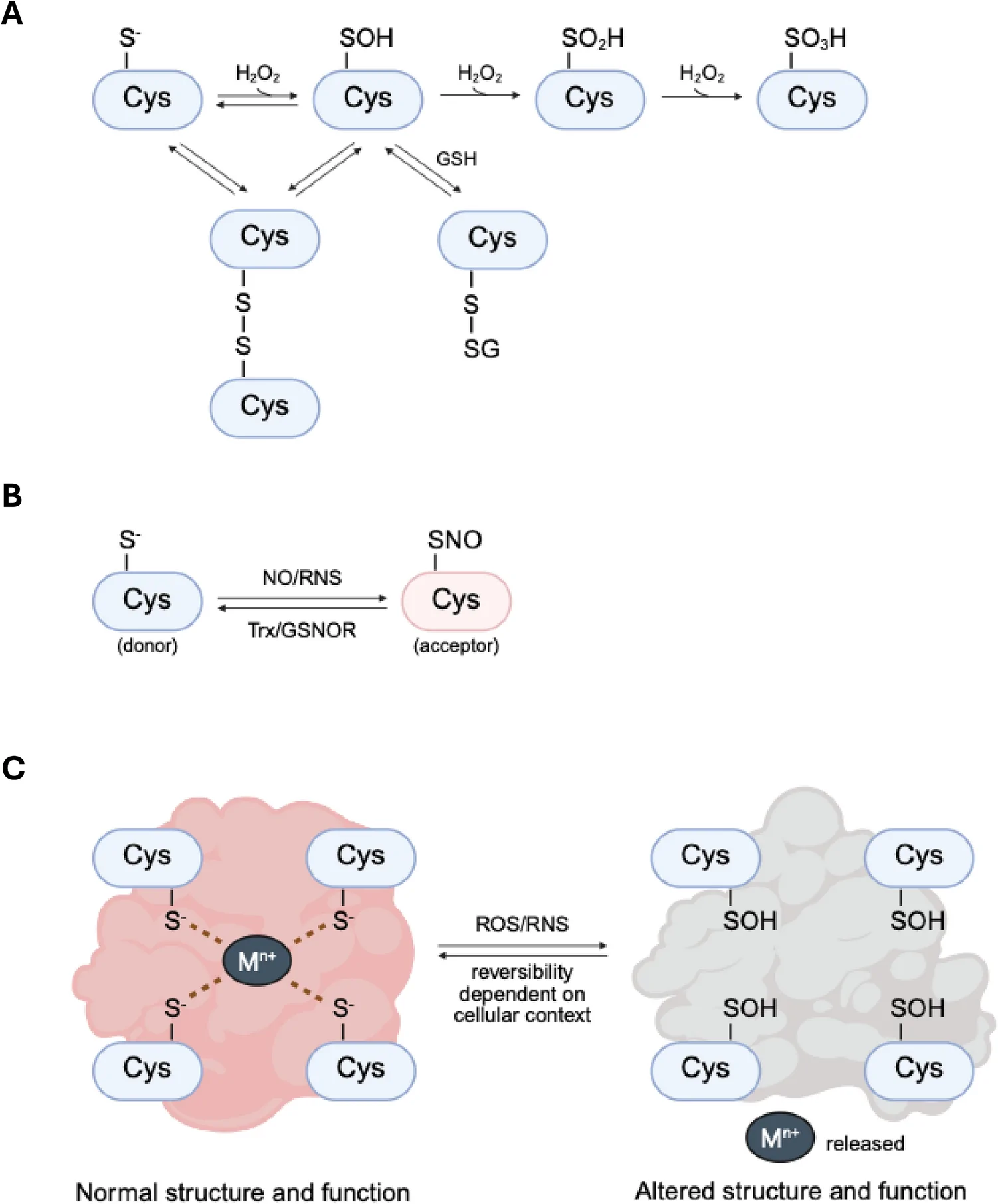
Cysteine modifications by reactive oxygen species, reactive nitrogen species, and metal-coordination. **A** Oxidative modifications of cysteine residues by hydrogen peroxide (H_2_O_2_). The cysteine thiolate (Cys-S^−^) is oxidised to sulfenic acid (Cys-SOH), a reversible intermediate (double-headed arrow). Cys-SOH can undergo reversible disulfide bond formation (Cys-S-S-Cys) with a second protein cysteine, or reversible *S*-glutathionylation (Cys-SSG) in the presence of glutathione (GSH). Under persistently elevated H_2_O_2_, Cys-SOH is further and irreversibly oxidised to sulfinic acid (Cys-SO_2_H) and sulfonic acid (Cys-SO_3_H), modifications associated with protein inactivation and degradation (single-headed arrows). **B** *S*-nitrosylation of cysteine residues by reactive nitrogen species (RNS). The cysteine thiolate (Cys-S^−^, donor) reacts reversibly with NO and related RNS to form *S*-nitrosocysteine (Cys-SNO, acceptor). Denitrosylation is catalysed by the thioredoxin system (Trx) and *S*-nitrosoglutathione reductase (GSNOR), regenerating the free thiolate. **C** Oxidation of metal-coordinating cysteine residues. Cysteine thiolates (Cys-S^−^) coordinate a metal ion (M^n+^) within the protein, maintaining normal structure and function. Oxidation by ROS/RNS converts the coordinating cysteines to Cys-SOH, releasing M^n+^ and altering protein structure and function. Reversibility is dependent on the cellular context. Figure created in BioRender

The reaction of thiolates with the oxidant hydrogen peroxide (H_2_O_2_) yields sulfenic acid (Cys-SOH) by *S*-sulfenylation, a reversible and critical intermediate in redox signalling (Fig. 1A). Sulfenic acid can undergo further oxidation to form intramolecular or intermolecular disulfide bonds (Cys-S-S-Cys) (Jo et al. 2015) or *S*-glutathionylation (Cys-SSG) (Kukulage et al. 2022). These are reversible PTMs that tightly regulate protein function under physiological conditions. However, under persistently elevated levels of H_2_O_2_, sulfenic acid may be further oxidised into sulfinic acid (Cys-SO_2_H) and sulfonic acid (Cys-SO_3_H), an irreversible modification that often inactivates the protein function, and marks the protein for degradation (Kukulage et al. 2022). In addition to oxygen-based modifications, *S*-nitrosylation of cysteine residues by the RNS nitric oxide (NO) provides another layer of reversible redox regulation (Fig. 1B) (Evangelista et al. 2013). Oxidation of thiols of cysteines involved in metal coordination can lead to the release of the bound metal ion, thereby modulating protein structure and function (Fig. 1C). Cysteinyl ligands are essential for metal ions such as zinc, iron, and copper, which are fundamental to the structural and functional integrity of many proteins, such as those that have zinc finger domains and iron-sulphur clusters (Maio and Rouault 2015; Oteiza 2012).

### Cysteines as sensors for electrophiles

**Fig. 2:**
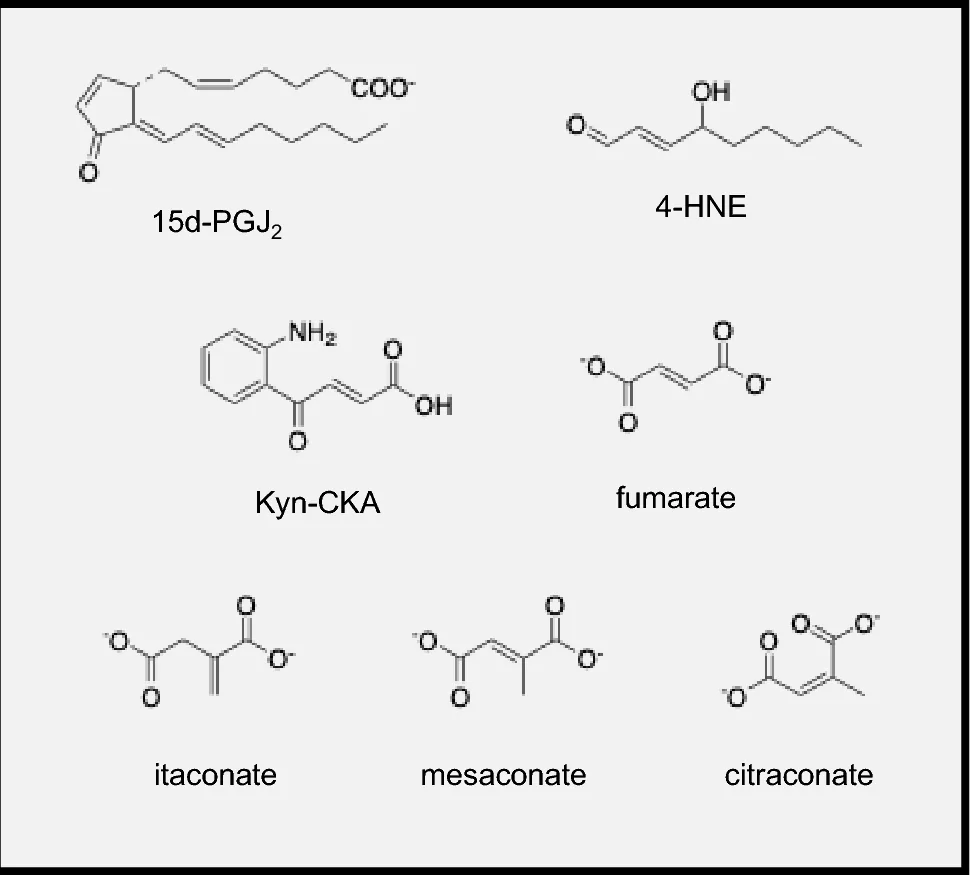
Chemical structures of representative endogenous reactive electrophilic species (RES). Endogenous RES capable of covalently modifying cysteine residues via Michael addition or nucleophilic substitution. The lipid-derived electrophiles 15-deoxy-Δ^12,14^-prostaglandin J_2_ (15d-PGJ_2_) and 4-hydroxynonenal (4-HNE) are produced during lipid peroxidation and inflammatory signalling. Kynurenine carboxyketoalkene (Kyn-CKA) is an electrophilic metabolite of the tryptophan-derived kynurenine. Fumarate, itaconate, citraconate, and mesaconate are mitochondria-derived dicarboxylate electrophiles with roles in innate immune signalling and metabolic stress responses. The electrophilic carbons in each structure undergo Michael addition with cysteine thiolates, forming covalent adducts that alter protein conformation and activity

Distinct from ROS/RNS-sensing, cysteine residues also serve as sensors for RES. These reactive species can covalently bind to thiols through nucleophilic substitution (e.g. alkylation) or Michael addition reactions, forming adducts that can alter protein conformation and activity, thereby modulating signalling cascades and cellular responses to stress (Long and Aye 2017). Prominent examples of endogenous RES are the lipid-derived cyclopentenone prostaglandin 15-deoxy-∆^12,14^-prostaglandin J_2_ (15d-PGJ_2_) and 4-hydroxynonenal (4-HNE), the electrophilic derivative of kynurenine, kynurenine carboxyketoalkene (Kyn-CKA), and the mitochondria-derived fumarate, itaconate, citraconate and mesaconate (Fig. 2). The electrophilic carbons of these RES can undergo Michael addition to cysteine residues. Such covalent modifications have been demonstrated for numerous protein targets, highlighting the ability of endogenously produced RES to modulate a plethora of cellular processes. Some examples include covalent cysteine modifications of the ubiquitin ligase substrate adaptor Kelch-like ECH-associated protein 1 (KEAP1) (Levonen et al. 2004; McMahon et al. 2010; Mills et al. 2018), phosphatase and tensin homolog (PTEN) (Suh et al. 2018), Harvey rat sarcoma viral oncogene homolog (H-RAS) (Oliva et al. 2003), multiple proteins in the nuclear factor-kappa B (NF-κB) signalling pathway (Chen et al. 2007; Donath et al. 2002; Rossi et al. 2000; Straus et al. 2000), and p53 (Kim et al. 2010).

Importantly however, whilst multiple proteins with reactive cysteines can undergo RES-mediated modifications, it is improbable that all of them react and are modified simultaneously. Moreover, bolus dosing of RES is highly likely to produce bystander effects. Indeed, it is now clear that the signalling events triggered by RES are dependent on both concentration as well as context. Elegant methodologies developed in the Aye laboratory (Long et al. 2022) have enabled the delivery of locale-specific RES, thus overcoming the limitations associated with bolus dosing for identifying RES-reactive proteins, such as bias towards abundant and/or cysteine-rich proteins or missing proteins that are exquisitely redox sensitive. The T-REX (Targetable Reactive Electrophiles and oXidants) methodology allows for precise targeting of specific proteins in specific subcellular environments in living cells and organisms. The G-REX (Global Reactive Electrophiles and oXidants) methodology extends the T-REX capabilities by working at a large scale, at the proteome level (Huang and Aye 2024). For localized responsivity to RES in the organismal context, Z-REX (Reactive Electrophiles and oXidants in live zebrafish embryos) and organ-specific (OS) Localis-REX in *C. elegans* methodologies were developed (Liu et al. 2024; Van Hall-Beauvais et al. 2022). The use of these methodologies has provided robust experimental evidence of the nuanced nature of RES signalling. Specific privileged electrophile sensors in response to 4-HNE were identified, such as C119 in the protein kinase AKT3 and C69 in the ubiquitin-conjugating protein UBE2V2, and C436 in the nuclear cap-binding protein subunit 1 (NCBP1) (Chang et al. 2026; Long et al. 2017; Zhao et al. 2018). Z-REX, in combination with a targeted knockdown approach, revealed that both zKEAP1a and zKEAP1b antagonized the antioxidant response at homeostatic conditions. However, only zKEAP1b was responsive to RES-mediated induction of the antioxidant response, and the lack of responsiveness of zKEAP1a was attributed to the fact that it bears an isoleucine at position 273 (numbering refers to the human protein) in place of the cysteine in zKEAP1b (Van Hall-Beauvais et al. 2022).

Notably, whilst Michael addition is the most common mechanism for electrophilic cysteine modification in biological systems, nucleophilic substitution reactions (such as SN2 or SNAr) are also possible. In these reactions, the cysteine thiolate (the nucleophile) attacks an electrophilic centre (often an aromatic or alkyl carbon) that bears a good leaving group, such as a halide (e.g. chloride, bromide, or iodide), and the covalent modification is accompanied by release of the leaving group. Although this reaction mechanism is a fundamental principle in organic and medicinal chemistry, the natural occurrence of thiol-based nucleophilic substitution in endogenous signalling is relatively rare. Nonetheless, these reactions are foundational for the development of cysteine-reactive probes and synthetic cysteine-targeted covalent inhibitors (Keeley et al. 2019; Yang et al. 2022). Together, these chemistries position cysteine as a prominent target for both endogenous control mechanisms and pharmacological strategies.

### KEAP1: a classical cysteine-based sensor

**Fig. 3:**
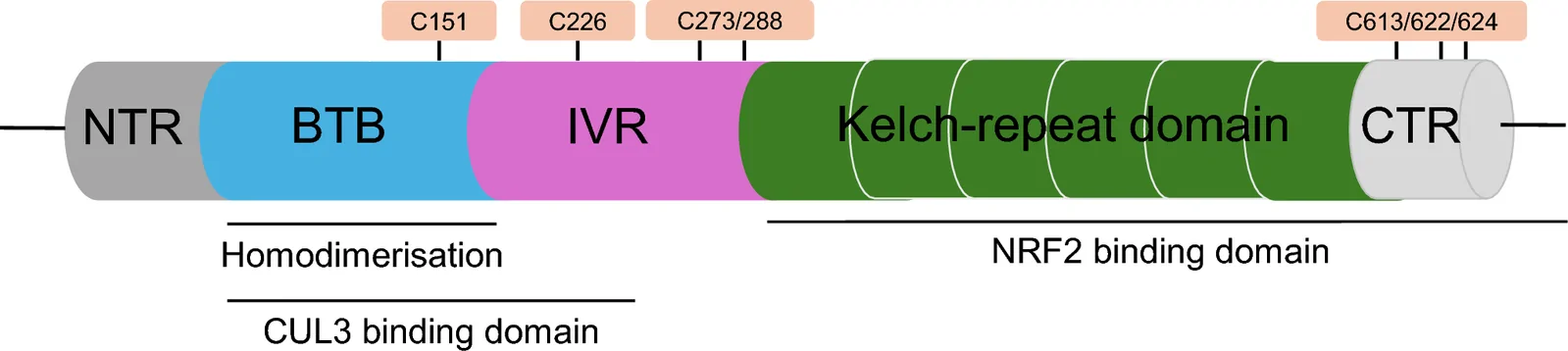
Domain architecture of KEAP1 and positions of key reactive cysteine residues. Schematic linear representation of the KEAP1 monomer showing its five functional domains: N-terminal region (NTR), BTB domain (Broad complex, Tramtrack and Bric-à-Brac), intervening region (IVR), double-glycine repeat/Kelch domain (DGR/Kelch), and C-terminal region (CTR). KEAP1 homodimerises through the BTB domain, which also mediates recruitment of CULLIN 3 (CUL3). The Kelch/DGR domains of the KEAP1 dimer provide the binding pockets for the Neh2 domain of NRF2. The positions of the key reactive cysteine residues are indicated: C151 (BTB; Class I sensor, preferentially modified by sulforaphane, fumarate derivatives, and omaveloxolone); C273 and C288 (IVR; Class II sensors, preferentially modified by 15d-PGJ_2_); C226, C613, C622, and C624 (Class IV sensors, mediating disulfide-based H_2_O_2_ sensing). Class III inhibitors (e.g. 4-HNE, 9-OA-NO_2_) require concerted sensing by C151, C273, and C288

KEAP1 is a homodimeric protein from the Broad complex, Tramtrack, and Bric-à-Brac (BTB)-Kelch family. Each KEAP1 monomer comprises five domains: an N-terminal region (NTR); a Broad complex, Tramtrack and Bric-à-Brac (BTB) domain; an intervening region (IVR); a double-glycine-repeat (DGR or Kelch) domain; and a C-terminal region (CTR) domain (Fig. 3) (Yamamoto et al. 2018). Endowed with highly reactive cysteines, KEAP1 serves as an intracellular cysteine-based sensor for a diverse range of chemical stressors, including RES, ROS and RNS. The ‘cysteine code’, whereby distinct cysteine residues have distinct chemical reactivities and spatial environments, enables KEAP1 to detect and discriminate among diverse reactive molecules in a context-dependent manner. Mass spectrometry and site-directed mutagenesis analyses have revealed the existence of distinct cysteine sensors and selectivity within KEAP1 (Dayalan Naidu and Dinkova-Kostova 2020; Dietz et al. 2005; Dinkova-Kostova et al. 2002). The functional significance of these cysteine residues has been examined in a variety of experimental systems, such as reporter co-transfection assays in mammalian cells, ectopic overexpression in zebrafish embryos, and transgenic complementation expression in mice (Eggler et al. 2009; Kobayashi et al. 2009; Takaya et al. 2012; Wakabayashi et al. 2004; Yamamoto et al. 2008; Zhang and Hannink 2003). Notably, these cysteines are critical for the function of KEAP1 as a stress sensor, but not for its function as an E3 ligase. An elegant study has shown that a Keap1 variant where 11 of its 27 cysteines (i.e. C151S/C226S/C257S/C273W/C288E/C319S/C434S/C489S/C613S/C622S/C624S) had been substituted still supported basal NRF2 turnover, but was insensitive to most cysteine-reactive electrophilic NRF2 inducers or H_2_O_2_ (Suzuki et al. 2019).

Based on differential cysteine modifications and/or mutagenesis studies demonstrating the requirement for specific KEAP1 cysteines for NRF2 de-repression, KEAP1 inhibitors are grouped into four classes (Suzuki et al. 2023). Class I inhibitors preferentially modify C151 in the BTB domain of KEAP1. With a pKa of 6.9, this cysteine is highly reactive at physiological pH due to its spatial proximity to basic amino acids, and is able to bind structurally diverse electrophiles, with a key hydrogen bond orienting the electrophilic carbon for a catalytic proximity effect (Schnell et al. 2025). Class I inhibitors include the electrophiles *tert*-butyl hydroquinone (tBHQ), sulforaphane (SFN), and kynurenine carboxyketoalkene (Kyn-CKA). C151 is also the site of modification by the clinically used monomethyl fumarate (MMF), dimethyl fumarate (DMF), diroximel fumarate, and omaveloxolone (as well as the related cyanoenone triterpenoids and their tricyclic and monocyclic derivatives). A recent study suggests that C151 is also involved in sensing the KRAS^G12C^-targeting drugs Sotorasib and Adagrasib (Baird et al. 2025). Whereas not affecting NRF2 binding, modification of C151 decreases the affinity of KEAP1 for Cullin 3 (CUL3), and consequently perturbs the KEAP1-mediated ubiquitination of NRF2 (Suzuki et al. 2025; Takaya et al. 2012). By contrast, VVD065, a recently discovered KEAP1 activator, increases the KEAP1-CUL3 interaction, promoting the degradation of NRF2 (Roy et al. 2025; Suzuki et al. 2025). Interestingly, some electrophiles have been shown to bind simultaneously to C151 and other amino acids, resulting in crosslinked high molecular weight species of KEAP1. Thus, the cyanoenone triterpenoid CDDO-Im forms a crosslink between C151 and Y85 (Suzuki et al. 2025). The glycolytic byproduct methylglyoxal forms a crosslink between C151 and R135 (Bollong et al. 2018). Formation of C151-dependent molecular bridges linking two KEAP1 monomers has also been observed upon treatment with electrophiles containing two Michael acceptor functionalities, such as *bis*(benzylidene)acetone and 4-ethyl-2,6-*bis*-pyridin-3-yl-methylene cyclohexanone (Bassil et al. 2026; Dayalan Naidu et al. 2026).

A notable feature of C151 is that its substitution with a serine or an alanine does not affect the ability of KEAP1 to repress NRF2 (He and Ma 2010; Zhang and Hannink 2003). Instead, it renders KEAP1 unresponsive to Class I inhibitors at concentrations which inactivate the wild-type protein both in vitro (Dayalan Naidu et al. 2018; Zhang and Hannink 2003) and in vivo (Gatbonton-Schwager et al. 2023). However, KEAP1 can still sense at least some of these inhibitors at higher inhibitor concentrations (Dayalan Naidu et al. 2018). These findings suggest the existence of an in-bult flexibility within the sensing mechanism and highlight its importance for providing cytoprotection, particularly in conditions when RES formation is high and could compromise survival.

Class II inhibitors show a preference towards C273/C288 within the intervening region (IVR) of KEAP1; these are exemplified by the electrophilic cyclopentenone prostaglandin (15d-PGJ_2_) (Levonen et al. 2004; Saito et al. 2015). Class III inhibitors, such as 9-nitro-oleic acid (9-OA-NO_2_), 4-hydroxynonenal (4-HNE), and sodium arsenite (NaAsO_2_), require concerted sensing by all three cysteines C151/C273/C288 for full activity (Saito et al. 2015). The redox signalling molecule H_2_O_2_ is classified as a Class IV inhibitor, where disulfide bond formation between any pair of four cysteines, i.e. C226, C613, C622, and/or C624, allows for sensing under oxidative conditions (Suzuki et al. 2019). By contrast, the gasotransmitter nitric oxide (NO) is inactive as NRF2 activator in cells expressing C151S-mutant KEAP1 (McMahon et al. 2010), based on which it is classified as a Class I inhibitor (Suzuki et al. 2023). It is also noteworthy that two possible mechanisms of activation of NRF2 by the gasotransmitter hydrogen sulfide (H_2_S_2_) have been proposed—direct modification of C151 (Yang et al. 2013) as well as indirectly, via the formation of H_2_O_2_ (Hourihan et al. 2013).

In addition to the cysteine-targeting covalent inhibitors of KEAP1 discussed above, non-electrophilic, non-covalent small molecules have been also developed—these bind to the Kelch domain of KEAP1, blocking its binding to the Neh2 domain of NRF2, and thus acting as KEAP1–NRF2 protein–protein interaction (PPI) inhibitors (Barreca et al. 2023; Wells 2015) and are sometimes referred to as Class V inhibitors (Suzuki et al. 2023). Although highly potent in vitro, due to their relatively large size, polarity, and metabolic instability, most of the early compounds were relatively weak inhibitors in cells and of limited efficacy in vivo. More recently however, metabolically stable PPI inhibitors have been reported, with potencies approaching the potencies of electrophilic KEAP1 inhibitors and demonstrated in vivo efficacy (Dayalan Naidu et al. 2022; Seedorf et al. 2023).

Notably, the electrophilic and the non-electrophilic compounds described above function as KEAP1 inhibitors, leading to NRF2 de-repression. By contrast, the recently described electrophilic compound VVD065 (and its derivative VVD130037, currently in clinical trials in patients with advanced solid tumors), a KEAP1 modulator which binds covalently to C151, acts as an allosteric molecular glue that promotes the formation of the KEAP1-CUL3 protein complex, thereby enhancing the degradation of NRF2 and decreasing the expression of NRF2-target genes (Kutsia et al. 2026; Roy et al. 2025; Suzuki et al. 2025).

### NRF2: the master regulator of the antioxidant response

NF-E2 p45-related factor 2 (NRF2) is a transcription factor that serves as the key regulator of cellular redox homeostasis and stress adaptation. First established as the principal activator of antioxidant responsive element (ARE)-driven genes (Itoh et al. 1997), NRF2 is now recognised as the central coordinator of defence against oxidative, electrophilic and xenobiotic stress and a drug target, activating cytoprotective genes that maintain redox homeostasis (Cuadrado et al. 2019; Dinkova-Kostova and Copple 2023; Zhang 2025). Through this cytoprotective transcriptional programme, NRF2 ensures metabolic stability, maintains proteostasis, and coordinates intracellular iron homeostasis, enhancing cellular fitness and resilience under stress conditions (Pajares et al. 2017).

**Fig. 4:**
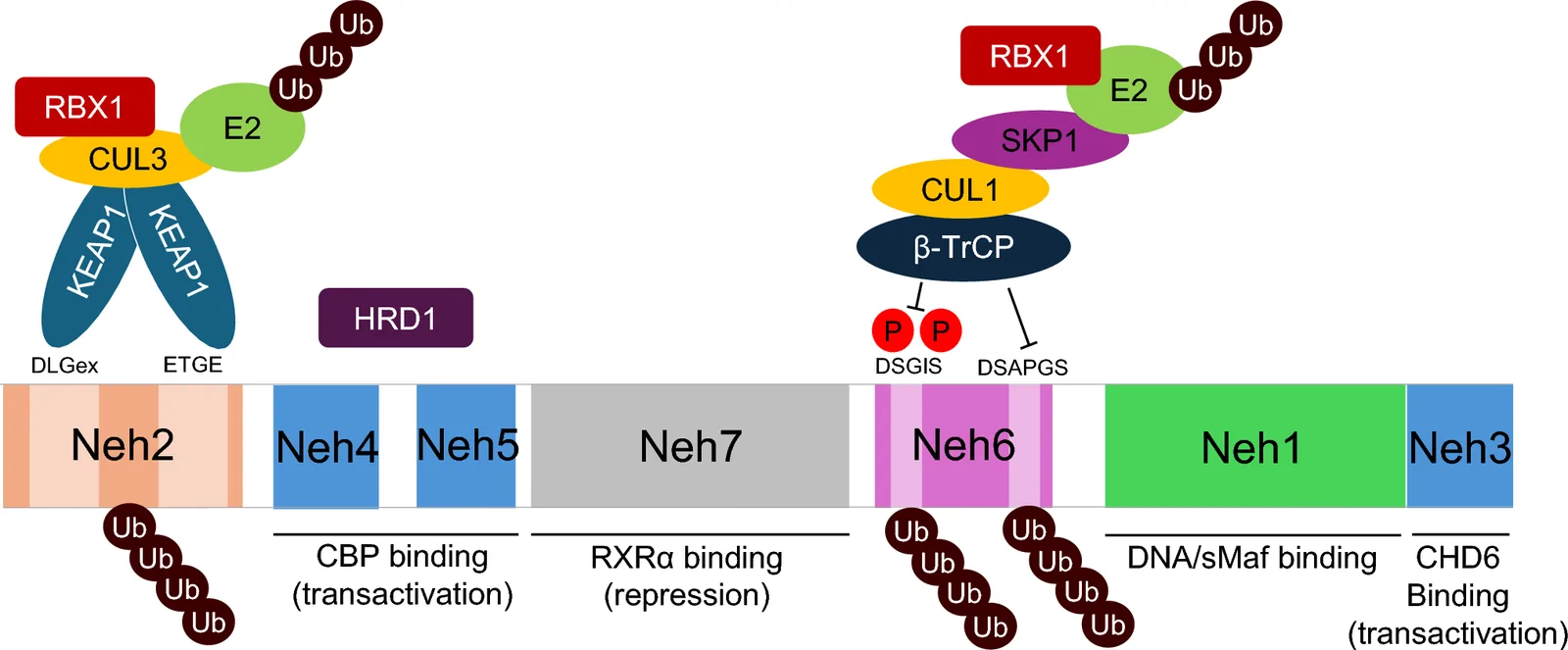
Domain architecture of NRF2 and functional annotations of the Neh domains. Schematic linear representation of NRF2 showing the seven Nrf2-ECH homology (Neh) domains. The Neh2 domain mediates bivalent interaction with the KEAP1 homodimer via the high-affinity ETGE motif (hinge) and low-affinity DLGex motif (latch). The Neh6 domain contains the DSGIS and DSAPGS phosphodegron motifs, which recruit the E3 ubiquitin ligase adaptor β-TrCP for KEAP1-independent NRF2 degradation in a GSK3-dependent manner. The Neh7 domain mediates transcriptional repression through interaction with RXRα. Neh3, Neh4, and Neh5 are transactivation domains; Neh4 and Neh5 cooperate to recruit the co-activators CBP and p300. The Neh1 domain contains the cap’n’collar (CNC)-bZIP motif, which mediates heterodimerisation with small MAF (sMAF) proteins and binding to antioxidant response elements (AREs). Nuclear localisation signals (NLS) and nuclear export signals (NES) regulate NRF2 nucleocytoplasmic shuttling

NRF2 is a modular protein composed of seven NRF2-ECH homology domains (Neh1-7), each with distinct functional roles (Fig. 4). The Neh1 domain of NRF2 contains the cap‘n’collar (CNC) basic-region leucine zipper (bZIP) motif, which is critical for its function as a transcription factor. This domain mediates heterodimerisation with the small musculoaponeurotic fibrosarcoma (sMAF) proteins, a prerequisite for recognising and binding to the ARE sequences (also known as CNC-sMAF binding elements, CsMBEs (Otsuki et al. 2016), in the regulatory regions of cytoprotective genes, and therefore transcriptional activity (Itoh et al. 1997, 1999). The Neh2 domain, which contains two critical motifs (ETGE and DLG), is responsible for binding KEAP1 (Itoh et al. 1997). Neh3, Neh4 and Neh5 function as transactivation domains, essential for NRF2-driven gene transcription. Notably, the deletion of the entire Neh5 domain has been shown to reduce the expression of endogenous NRF2 target genes, highlighting its role in transactivation (Zhang et al. 2007). The Neh4 and Neh5 domains cooperate to recruit important transcriptional co-activators, including CREB (cAMP Responsive Element Binding protein) Binding Protein (CBP) and p300, both of which possess histone acetyltransferase activity critical for chromatin remodelling and transcriptional activation. These interactions facilitate NRF2-driven gene expression by enabling chromatin accessibility at target AREs (Katoh et al. 2001). The Neh6 domain is serine-rich and contains two highly conserved degron motifs, DSGIS and DSAPGS, which mediate KEAP1-independent negative regulation of NRF2 via the recruitment of the E3 ubiquitin ligase adaptor β-transducin repeat-containing protein (β-TrCP) (Chowdhry et al. 2013; Rada et al. 2011, 2012). This provides an alternative degradation pathway independent of KEAP1, allowing for fine-tuned regulation of NRF2 stability. The Neh7 domain mediates transcriptional repression by direct interaction with retinoic X receptor alpha (RXRα), which prevents the recruitment of co-activators to the Neh4 and Neh5 domains, thereby inhibiting the transactivation activity of NRF2 (Wang et al. 2013). NRF2 also contains multiple nuclear localisation signals (NLS) and nuclear export signals (NES) within its structure, which allow the translocation of the transcription factor between the nucleus and the cytoplasm (Li et al. 2005, 2006; Theodore et al. 2008). This dynamic localisation is crucial for the rapid onset of the NRF2-mediated transcriptional response as well as for its attenuation.

NRF2 coordinates a broad transcriptional programme affecting drug metabolism, antioxidant response and lipid and iron metabolism, with target genes encompassing diverse biological pathways important for cellular defence (Hayes and Dinkova-Kostova 2014). Under NRF2 control, representative oxidoreductases include the aldo-keto reductases (AKR) family that reduce reactive carbonyls, and NAD(P)H:quinone oxidoreductase 1 (NQO1), a robust NRF2 target that catalyses the two-electron reduction of quinones to hydroquinones (Agyeman et al. 2011). Although global upregulation of cytochrome P450 enzymes (CYPs) is not a canonical NRF2 response, specific CYP genes such as *CYP1B1* and *CYP2B9* have been identified as NRF2 targets in a context-dependent manner (Hayes and Dinkova-Kostova 2014; Malhotra et al. 2010). Phase 2 drug metabolising enzymes generally facilitate conjugation reactions, coupling electrophilic intermediates with endogenous nucleophiles like glutathione (GSH), sulphate or glucuronic acid. For example, glutathione *S*-transferases (GSTs) and UDP-glucuronosyltransferases (UGTs), which increase metabolite solubility and promote excretion, are NRF targets (Chanas et al. 2002). Phase 3 transporters, mainly members of the ATP-binding cassette (ABC) family, actively export conjugated metabolites from the cells, completing detoxification. NRF2 induces several multidrug resistance-associated proteins (MRPs), organic anion transporting polypeptide 2 (OATP2), and P-glycoprotein (P-gp) in a gene- and tissue-specific manner (Wang et al. 2014).

In addition to regulating the basal and inducible expression of drug-metabolising enzymes, NRF2 also controls many key components in the endogenous antioxidant systems. At physiological levels, ROS, RES and RNS are effectively buffered by the NRF2-driven antioxidant program. The glutathione (GSH)-based antioxidant system is essential for maintaining redox homeostasis, and NRF2 is involved in the regulation of the GSH synthesis, utilisation and regeneration. NRF2 regulates the expression of glutamate-cysteine ligase catalytic (*GCLC*) and glutamate-cysteine ligase modifier (*GCLM*) subunits, which catalyse the rate-limiting step in GSH synthesis, as well as glutathione reductase (*GSR*), which regenerates reduced GSH from its oxidised GSSG form to maintain the antioxidant pool. NRF2 also regulates the gene expression of *SLC7A11*, which encodes the cystine/glutamate antiporter subunit (xCT) responsible for cystine import, which is then reduced intracellularly to cysteine, an essential substrate for GSH biosynthesis. Other antioxidant enzymes such as glutathione peroxidases (GPX) are also regulated by NRF2, and detoxify ROS by utilising GSH (Harvey et al. 2009; MacLeod et al. 2009). In addition, NRF2 has been implicated in the transcriptional regulation of superoxide dismutases (SOD1/2) and catalase, which catalyse the dismutation of superoxide and the decomposition of H_2_O_2_, respectively. The thioredoxin-based antioxidant system is also regulated by NRF2, which controls the expression of its key components thioredoxin 1 (*TXN1*), thioredoxin reductase 1 (*TXNRD1*), and sulfiredoxin 1 (*SRXN1*). These enzymes are essential for maintaining protein thiol redox balance by reducing oxidised protein thiols and peroxides, and thus controlling their activity (Hawkes et al. 2014).

In rapidly proliferating cells, NRF2 enhances the cellular reducing capacity by upregulating genes involved in the production of nicotinamide adenine dinucleotide phosphate (NADPH), including glucose-6-phosphate dehydrogenase (*G6PD*), malic enzyme 1 (*ME1*), and the enzymes in the pentose phosphate pathway (Mitsuishi et al. 2012). NRF2 also regulates the expression of isocitrate dehydrogenase 1 (*IDH1*) that catalyses the oxidative decarboxylation of isocitrate to α-ketoglutarate while generating NADPH. Together, these enzymes contribute to NADPH metabolism, redox balance and antioxidant defence (Wu et al. 2011). NADPH is a cofactor for multiple NRF2-regulated enzymes, and is essential for regenerating the GSH pool.

Beyond detoxification and antioxidant response regulation, NRF2 modulates both lipid and iron metabolism. In lipid metabolism, NRF2 regulates genes involved in fatty acid oxidation and lipid biosynthesis, and its upregulation has features of fasted metabolic state (Knatko et al. 2020). Acetyl-CoA thioesterases (ACOTs) regulate the pool of fatty acyl-CoA and free fatty acids, while acetyl-CoA oxidases (ACOXs) catalyse peroxisomal β-oxidation of long-chain fatty acids during fatty acid degradation. Carboxylesterases (CES1G, CES1H) and lipases (LIPH, PLA2G7, PNPLA2) mediate lipid mobilisation by hydrolysing triglycerides and phospholipids and contribute to lipid remodelling. NRF2 also induces key transcription factors, such as peroxisome proliferator-activated receptor gamma (PPAR-γ) and sterol regulatory-element binding protein-1c (SREBP1c), which drive lipogenesis and fatty acid uptake (Wu et al. 2011).

NRF2 plays a crucial role in iron and heme homeostasis by coordinating the network of genes involved in iron storage, heme synthesis and heme degradation to protect against iron-induced oxidative stress. It induces the expression of ferritin subunits (*FTH1*, *FTL1*), which sequesters free intracellular iron, thus preventing formation of potentially harmful ROS (Chorley et al. 2012). The synthesis and degradation of ferritin are orchestrated through the HECT and RLD domain-containing E3 ubiquitin protein ligase 2, HERC2, the autophagosome-lysosome fusion mediator vesicle-associated membrane protein 8 (VAMP8), and the selective cargo receptor nuclear receptor coactivator 4 (NCOA4), modulating the intracellular labile iron pool (LIP) and influencing susceptibility to iron-mediated cell death/ferroptosis (Anandhan et al. 2023). Simultaneously, NRF2 upregulates heme oxygenase-1 (HMOX1), which catalyses the breakdown of heme into biliverdin, free iron, and carbon monoxide, mitigating heme toxicity and enabling iron recycling (Alam et al. 1999). The resultant biliverdin is then converted by biliverdin reductases (BLVRA and BLVRB) into bilirubin, a potent antioxidant that further supports cellular redox balance. Additionally, NRF2 regulates ferrochelatase (FECH) to ensure proper heme biosynthesis by inserting iron into protoporphyrin IX within mitochondria (Wu et al. 2011). The lysosomal membrane-bound transporter heme-responsive gene 1 (*HRG1*, also known as *SLC48A1*), essential for heme-iron recycling, is also regulated by NRF2 (Kerins and Ooi 2018). Together, these interconnected processes coordinated by NRF2 safeguard cells from iron-driven oxidative damage and contribute to resistance to ferroptosis, underscoring the pivotal role of NRF2 in linking the antioxidant defence with iron metabolism.

### Regulation of NRF2

**Fig. 5:**
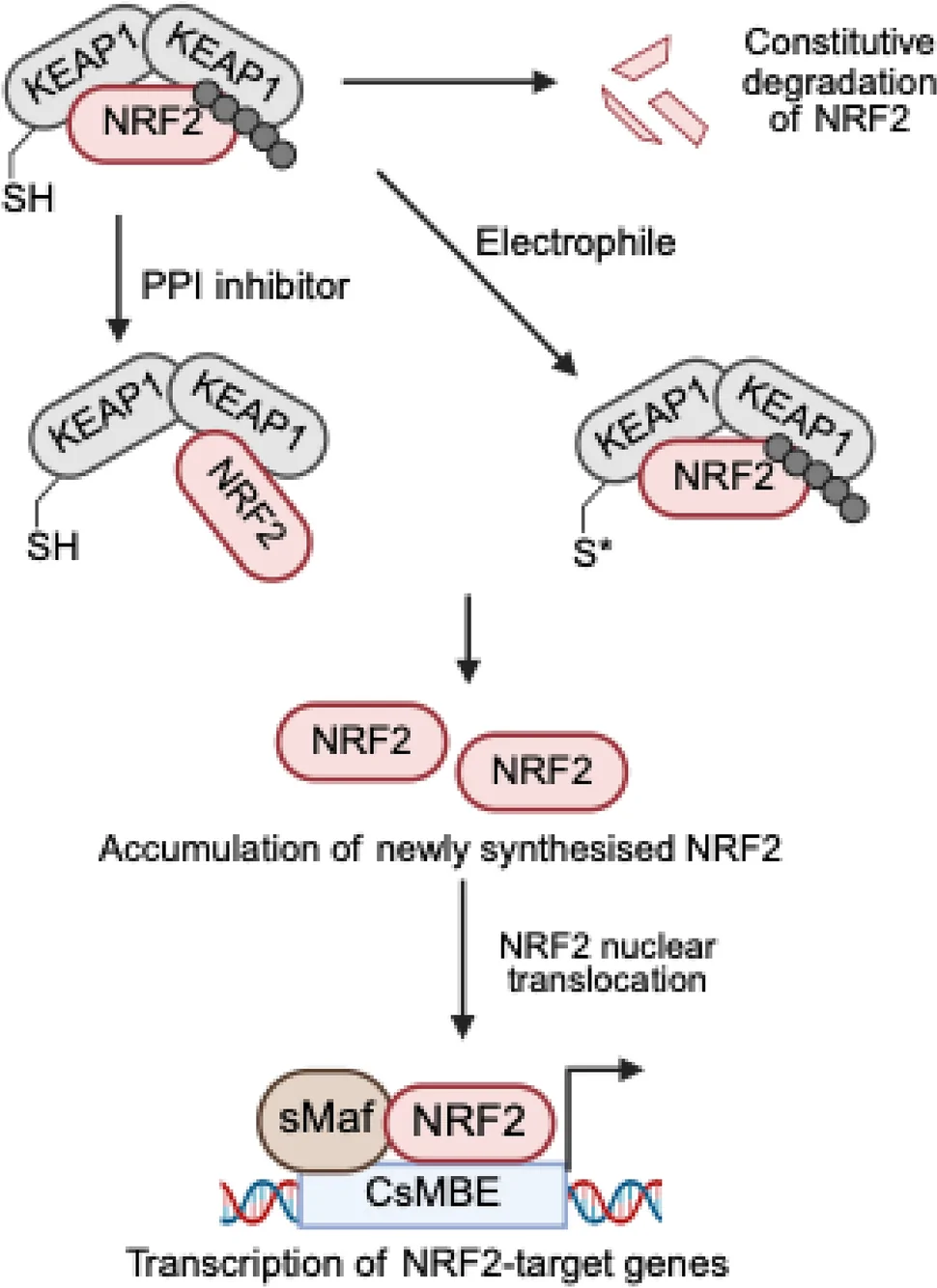
Mechanisms of NRF2 activation downstream of KEAP1. Under homeostatic conditions, the KEAP1 homodimer engages NRF2 through the ETGE and DLGex motifs of the Neh2 domain, facilitating the CUL3-mediated polyubiquitination (dark grey circles) and 26S proteasomal degradation of NRF2. Covalent modification of KEAP1 reactive cysteines by electrophiles (S* denotes modified cysteine) decreases the KEAP1–CUL3 interaction, thereby preventing NRF2 ubiquitination, but without disrupting KEAP1–NRF2 binding. Non-covalent protein–protein interaction (PPI) inhibitors competitively displace NRF2 from the Kelch domain binding pocket. In both activation routes, newly synthesised NRF2 escapes degradation, accumulates, and undergoes nuclear translocation, where it heterodimerises with a sMAF protein. The heterodimer binds to antioxidant response elements (AREs, also known as CNC-sMaf binding elements, CsMBEs), in the regulatory regions of NRF2-target genes, driving their transcription. Figure created in BioRender

Under homeostatic conditions, NRF2 is maintained at a low level by constitutive polyubiquitination and subsequent 26S proteasomal degradation mediated by the E3 ubiquitin ligases CRL3^KEAP1^, CRL4^DCAF11^, SCF^β-TrCP^, and HRD1, of which CRL3^KEAP1^ is the principal negative regulator of NRF2 (Hayes et al. 2025). KEAP1 homodimerisation and CUL3 recruitment occur through the BTB domain of KEAP1, and the Kelch domain of each subunit provides a binding pocket for the Neh2 domain of NRF2 (McMahon et al. 2006). In the canonical ‘hinge-and-latch’ arrangement, the KEAP1 dimer engages NRF2 bivalently via the high-affinity ETGE motif (hinge) and low-affinity DLGex motif (latch) (Tong et al. 2007). Förster resonance energy transfer (FRET)-based multiphoton fluorescence lifetime imaging (FLIM) of live cells and in vitro NMR spectroscopy titration experiments have validated this model by showing that incubation with non-electrophilic non-covalent KEAP1-NRF2 PPI inhibitors and phosphorylated p62 (also known as sequestosome-1) causes latch dissociation, whereas electrophilic cysteine-targeting covalent KEAP1 inhibitors do not disrupt KEAP1–NRF2 binding (Bertrand et al. 2015; Horie et al. 2021), but decrease the KEAP1–CUL3 interaction (Suzuki et al. 2025). Consequently, newly synthesised NRF2 accumulates and translocates to the nucleus to activate ARE/CsMBE-driven transcription (Fig. 5).

In addition to small molecule inhibitors of KEAP1, its interactions with other proteins can regulate NRF2. Under autophagy deficiency, p62 accumulates and becomes phosphorylated at S349 (numbering in the human protein) within its KEAP1 Interaction Region (KIR). The phosphorylation of the STGE motif confers high similarity to the ETGE motif in NRF2, thus increasing the affinity for the Kelch domain of KEAP1, competing against NRF2 binding (Horie et al. 2021; Ichimura et al. 2013; Komatsu et al. 2010). Notably, p62 is itself an NRF2 target gene, creating a positive-feedback loop that sustains NRF2 signalling under conditions of impaired autophagic flux (Jain et al. 2010; Komatsu et al. 2010).

NRF2 stability is also controlled by KEAP1-independent mechanisms. The Neh6 domain contains two motifs, DSGIS and DSAPGS, which create a phosphodegron and recruit the E3 ubiquitin ligase SCF^β-TrCP^ to promote NRF2 ubiquitylation and subsequent proteasomal degradation (Rada et al. 2011). The ability of DSGIS to serve as a degron is affected by the activity of glycogen synthase kinase 3 (GSK3) and the activity of a priming kinase. GSK3 phosphorylates serine residues within the DSGIS motif, converting it into a high-affinity phosphodegron for β-TrCP, whereas the DSAPGS binds in a largely phosphorylation-independent manner (Chowdhry et al. 2013; Rada et al. 2011). Accordingly, constitutively elevated GSK3 activity inhibits Nrf2 activity, while inhibition of GSK3 stabilises NRF2. Notably, dual inhibitors of both KEAP1- and GSK3-mediated NRF2 degradation, such as tBHQ and 6-(methylsulfinyl)hexyl isothiocyanate (hexaraphane), have been reported (Garcia-Yague et al. 2025; Hayes et al. 2015).

**Fig. 6:**
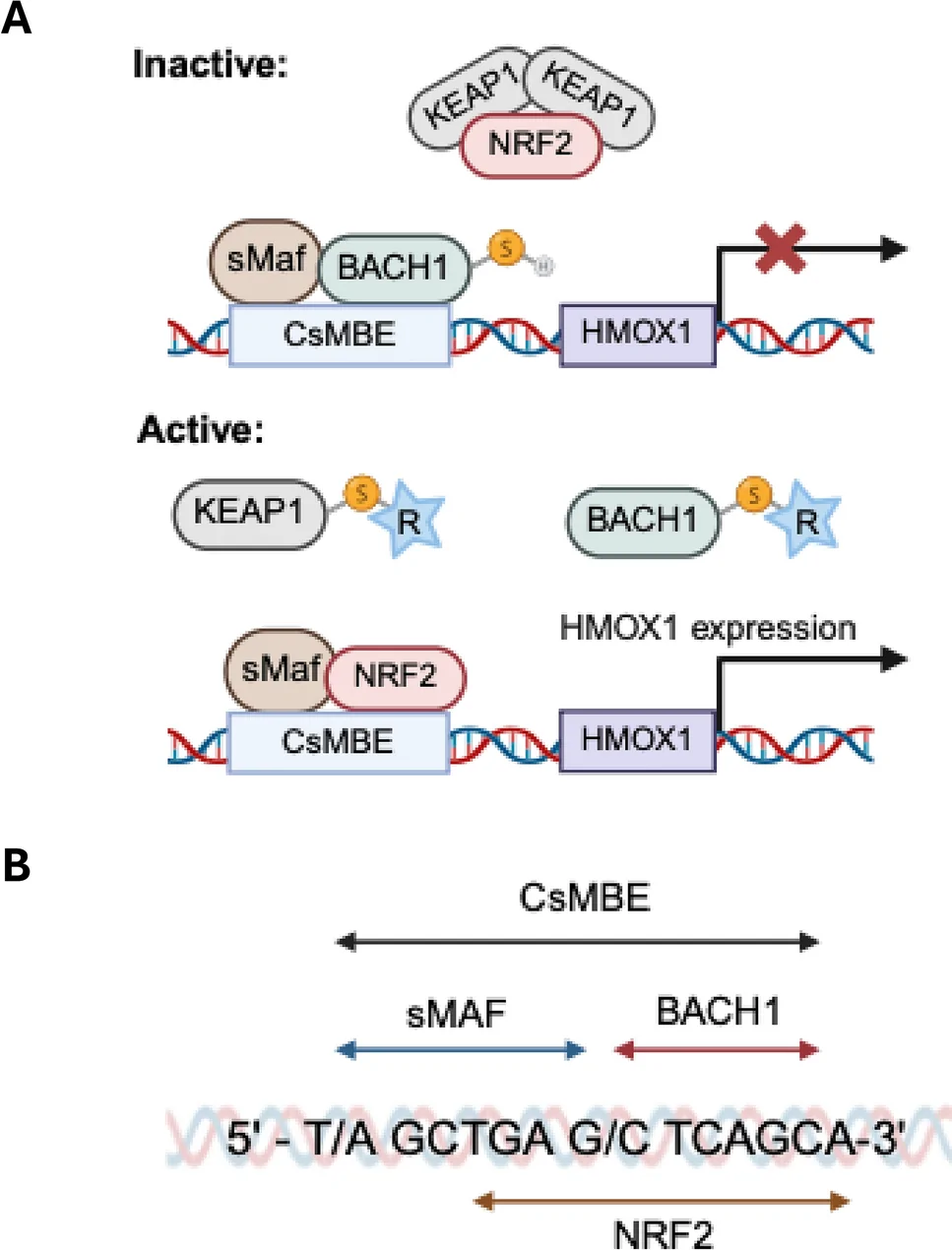
Competition between BACH1 and NRF2 at CsMBEs regulates *HMOX1* expression. **A** Inactive state (top): at homeostatic heme levels, BACH1 forms a repressor complex with a sMAF protein and occupies CsMBEs upstream of the *HMOX1* gene, blocking its transcription (red cross). Concurrently, KEAP1 maintains NRF2 at low levels through mediating its continuous ubiquitination and proteasomal degradation. Active state (bottom): under NRF2-activating conditions, KEAP1 reactive cysteines (S) are modified (R), impairing NRF2 degradation. BACH1 is displaced from the CsMBE by accumulated NRF2/sMAF complexes, enabling transcriptional activation of *HMOX1* and downstream HMOX1 protein expression (arrow). **B** Schematic of the CsMBE consensus sequence shared by BACH1/sMAF and NRF2/sMAF complexes, illustrating the competitive binding that underlies gene-selective regulation of the antioxidant response. Figure created in BioRender

At the transcriptional level, competition with other transcription factors further affects NRF2 activity. A key example is the crosstalk with NF-κB, where the subunit p65 competes with NRF2 for the limiting co-activators CBP/p300, a form of co-activators ‘squelching’ that reduces CBP/p300 availability at CsMBE-containing promoters (Kim et al. 2013; Liu et al. 2008). Transcription factor BTB and CNC homology 1 (BACH1) (discussed in detail in the sections below) acts as a gene-selective repressor by competing with NRF2 at DNA binding sites (Hushpulian et al. 2025; Igarashi et al. 2017; Pagnotta et al. 2022), thereby limiting induction of certain transcriptional targets, most notably *HMOX1* (Fig. 6) (Sun et al. 2002).

### The NRF2 antagonist BACH1

**Fig. 7:**
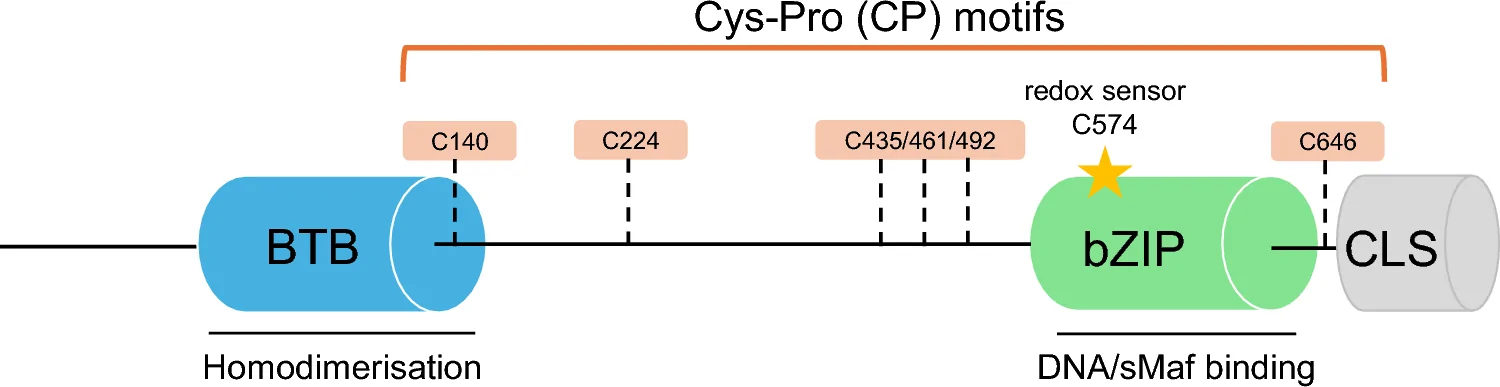
Domain architecture of BACH1 and positions of the cysteine-proline motifs. Schematic linear representation of BACH1, comprising an N-terminal BTB domain (which shares structural similarity with KEAP1) and a C-terminal bZIP domain (analogous to NRF2), connected by intrinsically disordered linker regions. Six cysteine-proline (CP) dipeptide repeats are distributed within the disordered linkers: CP1 (C140) and CP2 (C224) are located downstream of the BTB domain; CP3 (C435), CP4 (C461), and CP5 (C492) are clustered upstream of the bZIP domain; CP6 (C646) follows the bZIP domain. C435, C461, C492 and C646 are essential for heme-mediated regulation of BACH1 nuclear export and proteasomal degradation. C574 in the bZIP domain is a separate redox-sensitive cysteine; its modification by electrophiles or thiol-oxidising agents impairs DNA binding and induces cytoplasmic translocation of BACH1. CLS, cytoplasmic localization signal

Like NRF2, BTB and CNC homology 1 (BACH1) forms heterodimers with small MAF (sMAF) proteins. A recent biochemical study has shown that BACH1/MAFG and NRF2/MAFG heterodimers bind CsMBEs with similar affinities and can displace each other from pre-formed DNA-bound complexes (Tossounian et al. 2025). BACH1 is a modular protein, comprising an N-terminal BTB domain (which shares structural similarity with KEAP1) and a C-terminal bZIP domain (analogous to NRF2) (Fig. 7). The BTB domain adopts a conserved fold structure that can assemble into homodimers or higher-order assemblies. Although DNA recognition and sMAF heterodimerisation are governed by the bZIP domain, the BTB domain contributes to the multivalent complex formation with sMAFs on clustered CsMBEs.

**Fig. 8:**
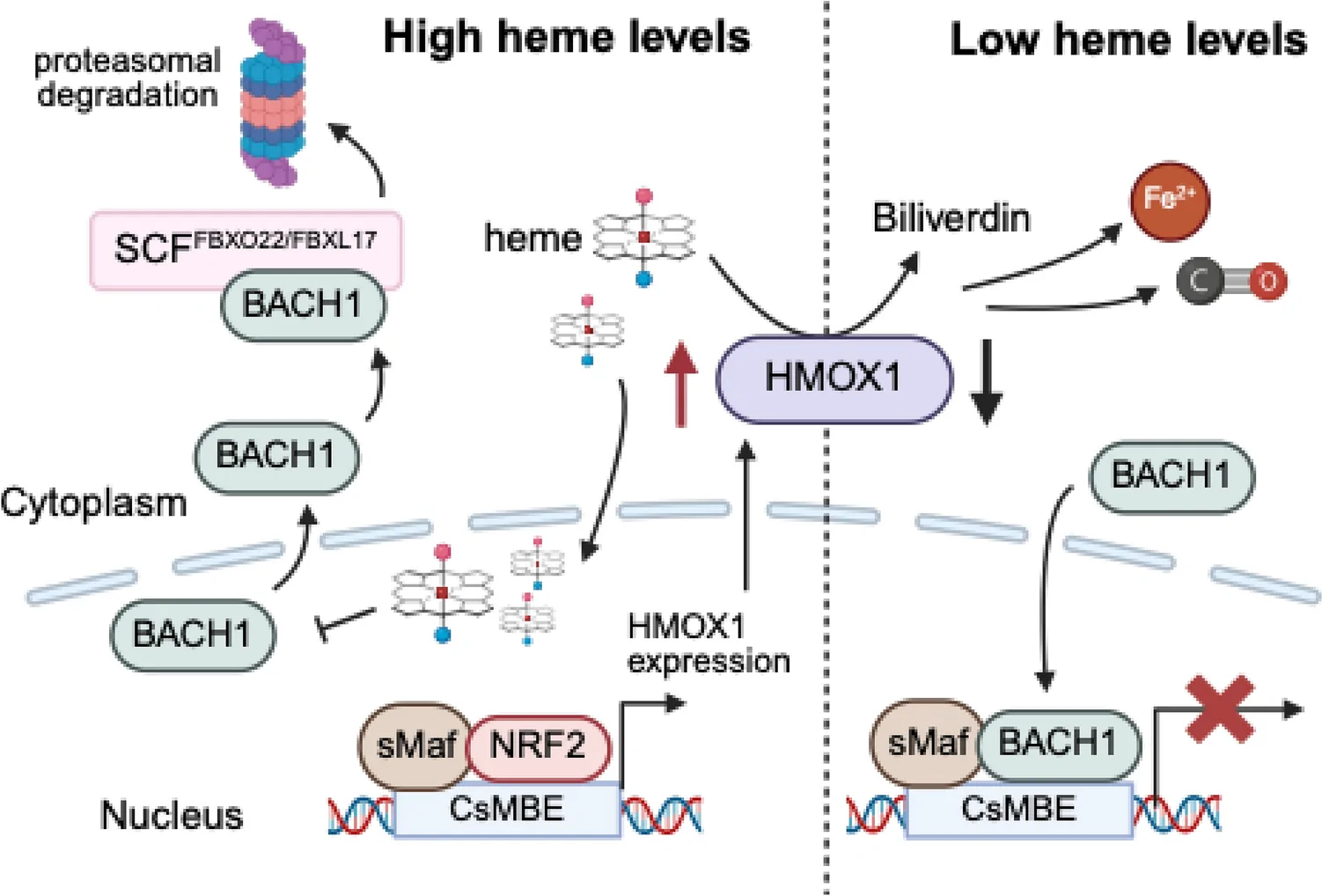
Heme-dependent regulation of BACH1 protein stability, localisation, and transcriptional activity. At low intracellular heme levels (left), BACH1 localises to the nucleus, forms heterodimers with sMaf proteins, and occupies CsMBEs at target gene loci (e.g. *HMOX1*), repressing their transcription. Upon elevation of intracellular heme (right), heme binds to the CP/HRM motifs of BACH1, inducing a conformational change that exposes a CRM1-dependent nuclear export signal (NES). BACH1 is exported from the nucleus to the cytoplasm, where it is sequestered at microtubules and becomes a substrate for polyubiquitination by the E3 ubiquitin ligases SCF^FBXO22^ and SCF^FBXL17^, followed by 26S proteasomal degradation. Derepression at the CsMBE permits NRF2/sMAF complexes to drive ARE-dependent transcription of *HMOX1*, resulting in HMOX1 protein expression. HMOX1 catalyses the degradation of heme to biliverdin, carbon monoxide (CO), and free iron (Fe^2+^), thereby decreasing intracellular heme levels and re-establishing conditions permissive for BACH1 nuclear re-accumulation, completing a negative feedback loop that maintains heme homeostasis. Figure created in BioRender

Between the BTB and bZIP domains are intervening discorded segments that lack stable secondary structure, providing the flexibility needed for the dynamic assembly and disassembly of the repressor complex. Six cysteine-proline (CP) dipeptide repeats are distributed within the intrinsically disordered linkers: two downstream of the BTB domain, three clustered upstream of the bZIP region and one following it (Fig. 7). Four of these CP motifs are essential for heme-mediated regulation of BACH1 (Fig. 8) (Ogawa et al. 2001). At low intracellular heme levels, BACH1 forms heterodimers with sMAFs and binds to the CsMBEs, antagonising NRF2/sMAF activator complexes at shared *cis*-elements. Upon heme binding, BACH1 undergoes a conformational change that exposes the CRM1-dependent nuclear-export signal, thereby promoting the nuclear export of BACH1 (Hira et al. 2007; Suzuki et al. 2004). Cytosolic BACH1 is sequestered at microtubules (Yamasaki et al. 2005), and becomes a substrate for polyubiquitination and rapid proteasomal degradation (Zenke-Kawasaki et al. 2007). This process releases repression of BACH1 target genes, permitting transcriptional activation by other transcription factors, such as NRF2. Direct target genes are considered those for which the CsMBEs have been mapped, BACH1 occupancy has been shown by chromatin immunoprecipitation (ChIP), and where reciprocal expression changes are observed upon heme treatment or BACH1 knockout/overexpression (Warnatz et al. 2011).

A core set of high-confidence BACH1 target genes regulate cellular heme and iron metabolism. The canonical target *HMOX1* contains multiple BACH1-binding motifs across the promoter and enhancer regions; these clustered CsMBEs provide high-affinity binding sites for BACH1 complexes, although only two of these sites have been shown to be reciprocally bound by both BACH1 and NRF2 (Sun et al. 2002; Warnatz et al. 2011). Similarly, genes encoding ferritin subunits (FTH1 and FTL) are repressed by BACH1 at baseline level, with induction observed following BACH1 knockout or heme addition. The combined induction of HMOX1 and ferritin promotes heme degradation and iron storage. This relieves the heme-mediated inhibition of BACH1, which in turn suppresses the expression of *HMOX1*, creating a negative feedback loop. However, persistent upregulation of *HMOX1* by other transcription factors, such as NRF2, can result in enhanced heme degradation and hyperactivation of BACH1. Thus, in lung cancer where NRF2 is frequently hyperactive, the levels of BACH1 are increased, leading to transcriptional upregulation of the expression of pro-metastatic genes and accelerated cell migration (Lignitto et al. 2019).

BACH1 has been reported to suppress SLC40A1 (ferroportin, the principal Fe^2^⁺ exporter), thereby increasing the labile iron pool and facilitating ferroptosis (Marro et al. 2010). Additional BACH1-sensitive transcripts include p62 (encoded by *SQSTM1*) and the transcription factor TFE3, both influencing cell cycle regulation. Notably, *SQSTM1* exhibits two distinct BACH1-binding peaks, reminiscent of *HMOX1*, indicating strong and direct regulation at this locus (Warnatz et al. 2011). Another way by which BACH1 affects ferroptosis is by binding to the promoter of *NOX4*, and increasing NOX4 expression, thereby promoting ROS generation (Jiao et al. 2026). Moreover, the deubiquitinate USP7 deubiquitinates and stabilizes BACH1, and BACH1 activates USP7, forming a positive feedback loop (Jiao et al. 2026). Overexpression of BACH1 in immortalized mouse embryonic fibroblasts (MEFs) increased the levels of the hepatokine fibroblast growth factor 21 (FGF21) by both inducing ferroptotic stress-mediated *FGF21* transcription and suppressing its autophagic degradation, through the transcriptional repression of p62 and LAMP2 (Nishizawa et al. 2024). The BACH1-mediated augmentation of ferroptosis has been implicated in a wide range of pathological processes, including neuropathic pain, traumatic brain injury, preeclampsia, pulmonary and myocardial ischemia–reperfusion injury, hypertension and sepsis (Bu et al. 2026; Cui et al. 2026; Gu et al. 2026; Jiao et al. 2026; Sun et al. 2026; Wu et al. 2026; Zou et al. 2025).

BACH1 modulates redox homeostasis, especially glutathione metabolism. In ChIP-seq analysis, the normalised reads of *GCLM* rank second among BACH1-bound loci (31.1 compared to 103.8 for *HMOX1*), indicating a strong baseline occupancy. In HEK293 cells, BACH1 depletion prominently upregulated *GCLM*, while *GCLC* remained largely unchanged. In HEK293 cells, one study showed that *SLC7A11* is downregulated after BACH1 knockdown, implying a potential activating role of BACH1 (Warnatz et al. 2011), whereas a subsequent study reported the accumulation of SLC7A11 protein in BACH1-depleted cells (Nishizawa et al. 2020). These findings suggest context- and cell-type-specific regulation of SLC7A11 by BACH1.

Among the BACH1 transcriptional targets is malic enzyme 1 (ME1), which catalyses malate-to-pyruvate conversion while generating cytosolic NADPH, a critical source of reducing equivalents for biliverdin reductase (Warnatz et al. 2011). Notably, while ChIP-seq analyses confirmed the association with BACH1-bound regions of several known target genes, SLC48A1 and FTL, two previously identified BACH1 target genes, did not change after BACH1 knockdown in HEK293 cells (Warnatz et al. 2011). Beyond metabolism, BACH1 has been implicated in calcium signalling and cell cycle control (Warnatz et al. 2011). Upregulation of inositol 1,4,5-trisphosphate receptor (ITPR2) enhances cytoplasmic calcium flux, promoting nuclear translocation of calmodulin (CALM1) and potentially influencing cell cycle and apoptosis. BACH1 also influence cytoskeletal dynamics and tumour biology, consistent with its established role in metastasis. Loss of BACH1 downregulates Vault RNA 1-1 (VTRNA1-1), a vault RNA involved in microtubule-mediated transport, while leaving other transport-related proteins largely unaffected, indicating selective regulation of the transport machinery. Among BACH1’s additional targets, HTRA3 (HtrA serine peptidase 3) has been identified as a regulator of cancer cell migration and apoptosis in lung cancer, and ZNF469 (zinc finger protein 469) is also a validated target that reflects basal BACH1 activity across multiple cancer types (Klenja-Skudrinja et al. 2025). The genomic distribution shows that approximately 55% of BACH1-binding regions contain a single CsMBE, while others harbour multiple motifs spaced 7 or 21 base pairs apart, supporting BACH1 homodimerization and cooperative DNA binding (Warnatz et al. 2011). These findings reveal the extensive regulatory network of BACH1 encompassing metabolism, antioxidant defence, calcium signalling, and autophagy.

### BACH1 regulation

Two E3 ubiquitin ligases, SCF^FBXO22^ and SCF^FBXL17^, are key regulators of the heme-dependent degradation of BACH1 (Lignitto et al. 2019; Tan et al. 2013). Recent studies have elucidated the complementary mechanisms for BACH1 recognition and ubiquitylation by these ligases (Cao et al. 2024; Goretzki et al. 2024). The domain-swapped β-sheet between β1 from one BTB domain and β5 of another BTB domain in BACH1 forms the crucial structural motif and functions as a quaternary degron recognised by FBXO22. In contrast, FBXL17 recognises BTB domains with destabilised domain swaps or in their monomeric form, through a degron localised at the dimer interface, which becomes exposed upon BTB domain dimer disassembly into monomers, and is subsequently ubiquitylated and degraded as part of the dimerisation quality control (DQC) system (Mena et al. 2020, 2018).

Remarkably, the regulation of BACH1 is yet another example of cysteine residues acting as a molecular switch for biological functions and modulating signalling pathways. An early study demonstrated that the mutation of C574 of BACH1 partially or completely inhibited the reactivation of transcriptional repression mediated by the RES 4-HNE or the thiol-oxidising agent diamide, respectively (Ishikawa et al. 2005). C574 is a conserved cysteine surrounded by basic amino acids in the bZIP domain of BACH1. Specifically, the substitution of this cysteine in the DNA-binding domain with serine (C574S mutant) rendered BACH1 unresponsive to diamide-induced derepression of BACH1 reporter activity. Moreover, the diamide-induced cytoplasmic translocation of a GFP-BACH1 fusion protein was also abolished in the C574S mutant. Most recently, it was shown that DNA binding by BACH1 is impaired in a C574-dependent, redox-sensitive manner (Tossounian et al. 2025).

The degradation of BACH1 can be induced by alkylating and *S*-nitrosylating agents. Notably, the combined treatment of NO and S-1-propenylcysteine (S1PC) induced BACH1 degradation within the nucleus. It has been proposed that cysteine residues could play an important role in these S1PC-mediated effects, since S1PC can interact with the *S*-nitrosothiols of BACH1 to influence its protein stability (Tsuneyoshi et al. 2019). Most recently, a structural study revealed that in vitro modifications of cysteine residues C107 and C122 in the BTB domain of BACH1 by bromo-1,1,1-trifluoroacetone (BTFA), a chemical that effectively mimics alkylation or *S*-nitrosylation, destabilised the degron for FBXO22 and induced a ligase switch to favour FBXL17-mediated degradation (Goretzki et al. 2024). Together, these studies highlight the role of cysteine modifications in the regulation of the localisation, stability and turnover of BACH1.

**Fig. 9:**
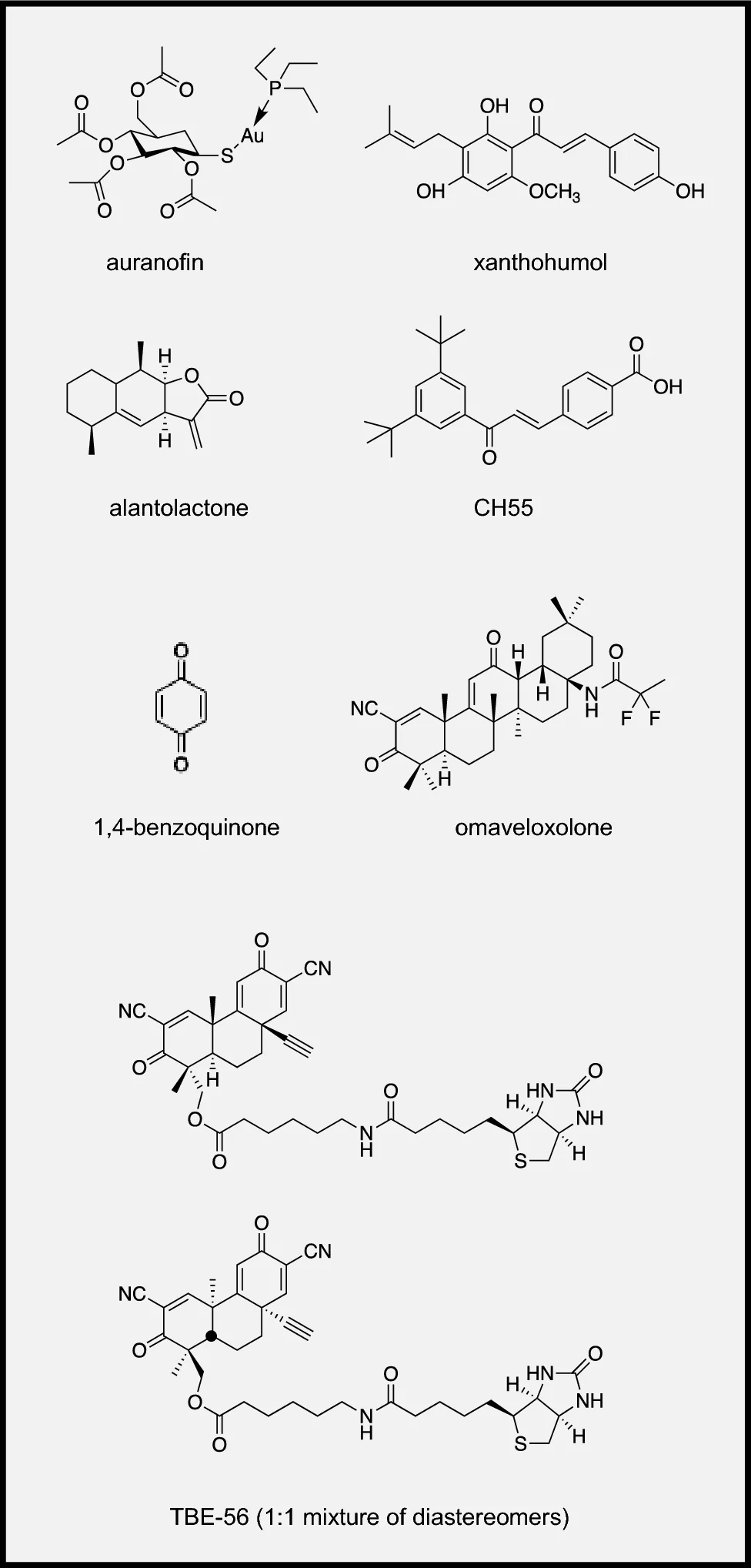
Chemical structures of compounds that dually inhibit KEAP1 and BACH1, converging on NRF2 activation. Auranofin, a clinically used, gold-containing thioredoxin reductase 1 (TXNRD1) inhibitor, induces KEAP1 oxidation consequent to TXNRD1 depletion, engaging the H_2_O_2_-sensing cysteines C613, C622, and C624 of KEAP1; its mechanism of BACH1 inhibition is proposed to occur via a similar oxidative route. Xanthohumol alkylates KEAP1 cysteines and reduces BACH1 protein levels. Alantolactone and CH55 were identified as BACH1 inhibitors in a high-throughput screen, and were also shown to activate NRF2. The electrophilicity of 1,4-benzoquinone is essential for its ability to inhibits both KEAP1 and BACH1. Omaveloxolone (a clinically used semi-synthetic oleanane triterpenoid) and TBE-56 (a biotinylated acetylenic tricyclic bis(cyanoenone) are dual KEAP1/ BACH1 inhibitors from the cyanoenone class

It is noteworthy that in addition to endogenously produced RES, such as NO and 4-HNE, several exogenous electrophiles have been shown to inhibit both KEAP1 and BACH1 (Fig. 9), activating NRF2. A recent high-throughput screen of > 2000 compounds identified four structurally distinct compounds (auranofin, xanthohumol, alantolactone and CH55) that inhibit BACH1-mediatd repression, and can also activate NRF2 (Ali et al. 2026). Auranofin is a potent inhibitor of TXNRD1 (Lu and Holmgren 2014). Depletion of TXNRD1 causes oxidation of KEAP1 in cells (Fourquet et al. 2010). Auranofin induces NRF2 in wild-type mice, but not in mice that lack the H_2_O_2_ sensor cysteines C613, C622 and C624 (Suzuki et al. 2019). Thus, it is possible that the ability of auranofin to inhibit BACH is also through BACH1 oxidation, consequent to TXNRD1 inhibition. Xanthohumol has been shown to alkylate KEAP1 cysteines (Luo et al. 2007). Although whether it could similarly alkylate BACH1 is unknown, xanthohumol decreases the protein levels of BACH1 (Ali et al. 2026). Similarly, quinones, such as 1,4-benzoquinone, inhibit both KEAP1 and BACH1, and the electrophilicity of these compounds is essential for their inhibitory effect (Abiko et al. 2011; Su et al. 2017; Wang et al. 2010). Among the dual KEAP1 and BACH1 inhibitors are the clinically used omaveloxolone, and other derivatives of the semi-synthetic oleanane triterpenoid 2-cyano-3,12-dioxooleana-1,9(11)-dien-28-oic acid (CDDO), as well as TBE-56, a biotinylated derivative of the acetylenic tricyclic bis(cyanoenone),(±)-(4bS,8aR,10aS)-10a-ethynyl-4b,8,8-trimethyl-3,7-dioxo-3.4b,7,8,8a,9,10, 10a-octahydrophenanthrene-2,6-dicarbonitrile (TBE-31) (Casares et al. 2022; Dinkova-Kostova et al. 2005; Luo et al. 2007; Moreno et al. 2022; Saito et al. 2013; Shekh-Ahmad et al. 2018). However, whereas active as KEAP1 inhibitors (Dinkova-Kostova et al. 2005, 2010), neither CDDO nor TBE-31 affect BACH1. Additionally, the naturally occurring triterpenoid oleanolic acid and its methyl ester, which are structurally similar to the CDDO derivatives, but lack the electrophilic carbon in ring A, are inactive (Casares et al. 2022; Dinkova-Kostova et al. 2005). Together, these findings suggest that, although not sufficient, the electrophilicity of these compounds is necessary for BACH1 inhibition, implicating the involvement of cysteine sensing in the underlying mechanism(s).

## Concluding remarks

The cysteine-based molecular switches KEAP1 and BACH1 exemplify the premise that non-enzymatic covalent cysteine modifications in proteins have functional consequences that are comparable to those caused by enzyme-catalyzed post-translational modifications of proteins involved in cell signalling. By antagonizing NRF2, KEAP1 and BACH1 ensure that the cell maintains a redox balance at homeostatic state. By virtue of their reactive cysteines, KEAP1 and BACH1 serve as molecular sensors for a wide range of ROS, RNS and RES. Crucially, both proteins are equipped with multiple reactive cysteines and thus have the ability to accommodate numerous structurally diverse compounds, providing a large degree of flexibility. The covalent modifications of the cysteine sensors of KEAP1 and BACH1 converge on NRF2 activation and the subsequent induction of the antioxidant response, which in turn ensures adaptation to the initial stimulus and re-establishes the homeostatic state.

## Data Availability

No datasets were generated or analysed during the current study.
